# Bioelectrical Impedance Methods for Noninvasive Health Monitoring: A Review

**DOI:** 10.1155/2014/381251

**Published:** 2014-06-17

**Authors:** Tushar Kanti Bera

**Affiliations:** Department of Computational Science and Engineering, Yonsei University, Seoul 120749, Republic of Korea

## Abstract

Under the alternating electrical excitation, biological tissues produce a complex electrical impedance which depends on tissue composition, structures, health status, and applied signal frequency, and hence the bioelectrical impedance methods can be utilized for noninvasive tissue characterization. As the impedance responses of these tissue parameters vary with frequencies of the applied signal, the impedance analysis conducted over a wide frequency band provides more information about the tissue interiors which help us to better understand the biological tissues anatomy, physiology, and pathology. Over past few decades, a number of impedance based noninvasive tissue characterization techniques such as bioelectrical impedance analysis (BIA), electrical impedance spectroscopy (EIS), electrical impedance plethysmography (IPG), impedance cardiography (ICG), and electrical impedance tomography (EIT) have been proposed and a lot of research works have been conducted on these methods for noninvasive tissue characterization and disease diagnosis. In this paper BIA, EIS, IPG, ICG, and EIT techniques and their applications in different fields have been reviewed and technical perspective of these impedance methods has been presented. The working principles, applications, merits, and demerits of these methods has been discussed in detail along with their other technical issues followed by present status and future trends.

## 1. Introduction

A living object such as an animal or plant is developed with cells and tissues arranged in three dimensional arrays. For example, the human body is a biological subject which is a very complex structure constructed by several living tissues [1] composed of the three-dimensional arrangement of human cells. The biological cells, containing intracellular fluids (ICF), cell membranes with or without cell wall, are suspended in the extracellular fluids (ECF) and show a frequency dependent behavior to an alternating electrical signal. Under an alternating electrical excitation, the biological cells and tissues produce a complex bioelectrical impedance or electrical bioimpedance [2–4] which depends on tissue composition and frequency of the applied ac signal [2–4]. Therefore the frequency response of the electrical impedance of the biological tissues is highly influenced by their physiological and physiochemical status and varies from subject to subject. Even the complex bioelectrical impedance varies from tissue to tissue in a particular subject and also varies with the change in its health status [5, 6] depending on the physiological and physiochemical changes occurred in the tissues health. Hence, the studies on complex bioimpedance of a tissue can provide a lot of information about its anatomy and physiology. Moreover, as the bioelectrical impedance of a body tissue depends on the signal frequency, the multifrequency studies on the electrical impedance of the biological tissues can potentially be used for the noninvasive investigations of their physiological or pathological properties.

Electrical impedance based noninvasive tissue characterizing techniques like bioelectrical impedance analysis (BIA) [7–21], electrical impedance spectroscopy (EIS) [22–32], electrical impedance plethysmography (IPG) [33, 34], impedance cardiography (ICG) [35–37], and electrical impedance tomography (EIT) [38, 39] are being used to study the frequency response of the electrical impedance of biological tissues. But EIS is found more popular in several fields of application compared to BIA, IPG and ICG as it provides the impedance variations over frequencies. Also, EIS has been studied for the noninvasive characterization of biological as well as nonbiological materials in frequency domain whereas BIA, IPG and ICG are used in biological fields only. On the other hand, the BIA, IPG, and ICG are applied on biological tissues and, generally, at a particular frequency. BIA, IPG, and ICG all are impedance analyzing techniques which provide the impedance values of the tissue sample as a lumped estimation, whereas the bioelectrical EIS calculates and analyzes the electrical impedance at different frequencies which enable us to obtain not only the impedance values of the tissue sample as a lumped estimation at a suitable frequency (generally, 50 kHz) but also it provides the information to understand several complex bioelectrical phenomena like dielectric relaxation and dielectric dispersions. The information about the dielectric relaxation [40–42] and *α*, *β*, and *γ* dispersions [40–42] enables us to analyze and understand the complex bioelectrical phenomena [40–42] occurring in cells and tissues under an alternating electric current signal. BIA, EIS, IPG, and ICG investigate the tissue properties by assessing the lump impedance parameters obtained from the boundary voltage current measurement. On the other hand, EIT provides a spatial distribution (2D or 3D) of the impedance profile of a domain under test using a set of boundary voltage-current data. Therefore, EIT is found with the potential of visualizing the tissue physiology and pathology in terms of tomographic images of the electrical impedance distribution, and hence it has been applied in several applications.

The paper has discussed about the BIA, EIS, IPG, ICG, and EIT techniques and reviewed their technical perspective along with their application in different fields. The working principle, applications, merits, and demerits of all these methods have been discussed in detail in this paper along with their other technical issues. The paper discusses about the present status, challenges, and future directions of the impedance methods.

## 2. Bioelectrical Impedance

### 2.1. Biological Tissues and Their Bioelectrical Impedance

The animals or plants are the living subjects which are developed with cells and tissues arranged in three-dimensional (3D) space. Animals and plants are developed with animal tissues and plant tissues, respectively. The animal tissues and the plant tissues are composed of the 3D arrays of animal cells and plant cells, respectively. Human body is a complex biological structure composed of several tissues [1] composed of the 3D arrays of cells. All the biological cells, such as animal cells and plant cells, contain intracellular fluids (ICF) and cell envelop (cell membrane for animal cells and cell membrane and cell wall for plant cells) and are suspended in extracellular fluids (ECF). The animal cells and plant cells suspended in extracellular fluids (ECF) show a different behavior to an alternating electrical signal producing a complex electrical impedance [2–4] which is called bioelectrical impedance or electrical bioimpedance.

The bioelectrical impedance depends on the tissue composition as well as the frequency of the applied ac signal [2–4], and hence, the frequency response of the bioelectrical impedance of the biological tissues not only depends on their physiological and physiochemical status but also it varies with applied signal frequency. Moreover the bioelectrical impedance varies from tissue to tissue and subject to subject, even if it varies for the measurement directions in anisotropic tissue structures. Even the complex bioelectrical impedance changes due to the variation in tissue structure, composition, and health status [5, 6]. Hence, by studying the impedance analysis of biological tissue, one can obtain a lot of information about the tissue anatomy and tissue physiology. Therefore, the complex electrical impedance analysis of biological tissues is found to be an efficient tool for noninvasive investigations of physiological or pathological status.

### 2.2. Origin of the Bioelectrical Impedance

The biological subjects are developed with biological tissues which are developed with biological cells arranged in a very complex three-dimensional arrays suspended in extracellular matrix called an extracellular fluids (ECF) [5]. In animal tissue, the cells are composed of intracellular fluids (ICF), cell membranes (CM) which are suspended in extracellular fluids (ECF) whereas the plant cells are composed of ICF, CM, and cell wall (CW) and are suspended in ECF.

**Figure 1:**
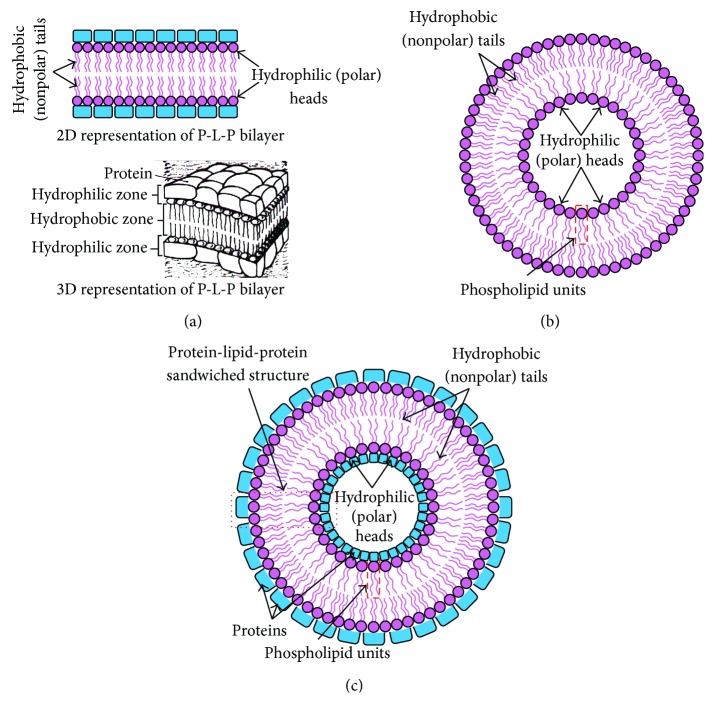
Cell membrane structure of the biological cells: (a) 2D and 3D model of P-L-P layers of a isolated cell membrane part, (b) 2D model of the lipid bilayers in cell membranes with hydrophilic (polar) heads and hydrophobic (nonpolar) tails, and (c) 2D model of the protein-lipid-protein sandwiched structure of a cell membrane.

ICF, CM, and ECF all are composed of different materials having their distinguished electrical properties, and therefore each of these cell and tissue components respond distinguishably to an alternating current signal. The intracellular fluids are composed of the cytoplasm and the nucleus. The cytoplasm and the nucleus are mostly made up of solution of proteins, different chemicals, salts, and waters, and hence these materials are electrically conducting. The extracellular fluids are also made up of electrically conducting materials. As the intracellular fluids and extracellular fluids in biological tissues are composed of ionic solution and the highly conducting materials, they provide highly conducting paths (low resistive paths) to the applied current signal [34]. But, the cell membranes (Figure 1) of the cells in a biological tissue are composed of electrically nonconducting lipid bilayers (Figure 1(a)) sandwiched between two conducting protein layers [35, 36] and form the protein-lipid-protein (P-L-P) structures. In the P-L-P structures (Figure 1(b)) the hydrophobic tails do not absorb the water and hydrophilic heads are attached to the protein layers. The P-L-P [35, 36] sandwiched structure (Figure 1(c)) provides a capacitance [34] to the applied alternating current signal and contributes to a capacitive reactance.

As a result, the overall response of the biological tissues to an alternating electrical signal applied to it, produces a complex bioelectrical impedance (*Z*_*b*_) [2–4] which is a function of tissue composition as well as the frequency of the applied ac signal [2–4]. The impedance *Z*_*b*_ is a complex quantity and varies with tissue structure, tissue composition, tissue health, and signal frequency. Therefore the impedance varies from subject to subject, tissue to tissue, and sometime with measurement direction within the same tissue. Even, the bioimpedance of a tissue may vary from other parts of the same tissue due to the variation in tissue composition, tissue physiology, and tissue pathology or tissue health.

### 2.3. Bioelectrical Impedance of Diseased Tissue

As the tissue physiology and pathology change with the tissue health, the bioelectrical impedance also varies from a healthy tissue to a diseased tissue. Thus the bioelectrical impedance changes with the variations in tissues health status [5, 6] such as swelling, disease, and infection. Similarly the impedance of a normal tissue is found more than a tumor or cancerous tissue as the cancerous tissue needs more blood to continue its rapid and uncontrolled growth which needs more blood. As the blood is good conductor of electricity, the cancerous tissue containing more blood shows a less impedance path to the electrical current.

### 2.4. Frequency Response of Bioelectrical Impedance

The frequency response of the bioelectrical impedance depends on their anatomical, physiological, and pathological status of the biological tissues. Therefore, the studies on the electrical bioimpedance of a tissue can provide a lot of information about its anatomy and physiology. As the response of the electrical bioimpedance varies with signal frequency, multifrequency impedance analysis provides more information about the tissue properties which help in the better tissue characterization. Therefore, the multifrequency impedance analysis of the biological tissues is found very promising for the noninvasive investigation of physiological or pathological status. As a result, a lot of research studies have been conducted on the single frequency impedance analysis as well as the multifrequency impedance analysis on biological tissues to investigate their physiological or pathological health status noninvasively for tissue characterization as well as for the diagnosis of a number of diseases.

## 3. Bioelectrical Impedance Analysis (BIA)

### 3.1. Impedance Measurement

Electrical impedance of a particular part of an object (volume conductor) is estimated by measuring the voltage signal developed across that body part by injecting a constant current signal to the object. Mathematically, the impedance (*Z*) is measured by dividing the voltage signal measured (*V*) by the current signal applied (*I*). *Z* is complex quantity and it will have a particular phase angle (*θ*) depending on the tissue properties. In electrical impedance measurement process, the bioelectrical impedance of a body tissue is measured by injecting a low amplitude low frequency alternating current (generally sinusoidal) to the tissue through an array of surface electrodes attached to the tissue surface (tissue boundary). The alternating current is applied to avoid the tissue damage, and hence the bioimpedance measurement is never conducted with the direct current signal.

In BIA, the bioimpedance *Z∠θ* is found as the transfer function of the SUT, and thus the *Z∠θ* is calculated by dividing the voltage data (*V∠θ*_1_) measured by applied current (*I∠θ*_2_) as shown in

(1) $$\begin{array}{c} Z∠\left(\theta \right)=\frac{V∠\left(\theta_{1}\right)}{I∠\left(\theta_{2}\right)}. \end{array}$$

### 3.2. Two-Electrode and Four-Electrode Methods

**Figure 2:**
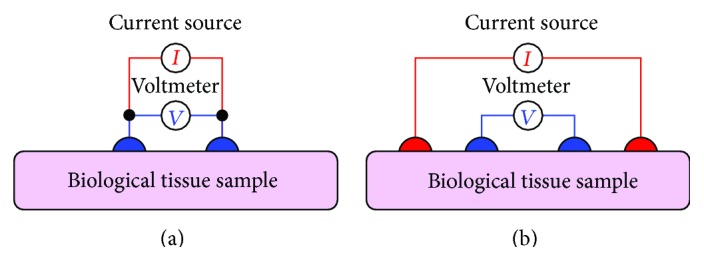
Bioelectrical impedance measurement in technique: (a) impedance measurement using two-electrode technique, (b) impedance measurement using four-electrode technique.

The bioimpedance measurement process is conducted by either the two electrode or four-electrode methods. In both the methods, the surface electrodes through which the current signal is injected are known as the current electrodes or the driving electrodes (as shown by the red colored electrodes in Figure 2) and the electrodes on which the frequency dependent ac potential (*V*(*f*)) is measured are called voltage electrodes or sensing electrodes (as shown by blue colored electrodes in Figure 2).

As the name tells, the two-electrode method (Figure 2(a)) uses only two electrodes for impedance measurement, and hence the current signal injection and voltage measurement are conducted with same electrodes. The two-electrode-method, therefore, suffers from the contact impedance problem and the measurement data contains the voltage drop due to the contact impedance. In the four-electrode method (Figure 2(b)), two separate electrode pairs are used for current injection and voltage measurements, and hence the four-electrode method is found as an impedance measurement method with a linear array of four electrodes attached to the SUT as shown in Figure 2(b). The four-electrode method injects a constant amplitude current signal to the SUT through the outer electrodes called current electrodes or the driving electrodes (red colored electrodes in Figure 2(b)) and the frequency dependent developed voltage signals are measured across two points within the current electrode through the inner electrodes called voltage electrodes or the sensing electrodes (blue colored electrodes in Figure 2(b)).

### 3.3. Bioelectrical Impedance Analysis

Bioelectrical impedance analysis (BIA) is a technique in which the body composition of a biological object is analyzed by measuring its bioelectrical impedance. Hence the bioelectrical impedance analysis measures the bioimpedance of the tissue which is produced inside the biological object when an alternating current tends to flow through it, and hence it is found as a function of tissue properties as well as the applied current signal frequency. As explained earlier, like other electrical impedance measurement techniques, in bioimpedance measurement methods, a constant sinusoidal current is injected to the subject under test (SUT), or a biological tissue sample, and the voltage developed is measured using four-electrode method [5] or four-probe method, and the electrical impedance of the SUT is calculated. In four-electrode method of bioimpedance measurement process, a constant amplitude alternating current is injected through two electrodes (outer electrodes) called current electrodes and the voltages are measured on other two electrodes (inner electrodes) named as voltage electrodes as shown in Figure 2 [5]. BIA determines the bioelectrical impedance of a particular body part attached with surface electrodes [5]. Bioelectrical impedance of a subject can be used to calculate an estimate of the body composition.

A bioelectrical impedance analysis (BIA) [7–14] is a technique in which the body composition [14–21] of a biological object is analyzed by measuring its electrical impedance called bioelectrical impedance or electrical bioimpedance. Dr. William Mills, M.D., an Admiral in the US Navy, initiated the study on BIA in Mount McKinley, Alaska, in 1981, to assess the hydration status of soldiers in high altitude cold weather environments. The paper published by Hoffer et al. [7] in 1969 indicated a procedure to predict the total body water by measuring the hand to foot whole body BIA and with the encouragement of Jan Nyboer the Mount McKinley soldier hydration project was started. Shortly thereafter Lukaski et al. [8] at the USDA in Grand Forks, ND, published the first to a paper on BIA and body composition.

### 3.4. Body Compositions

**Figure 3:**
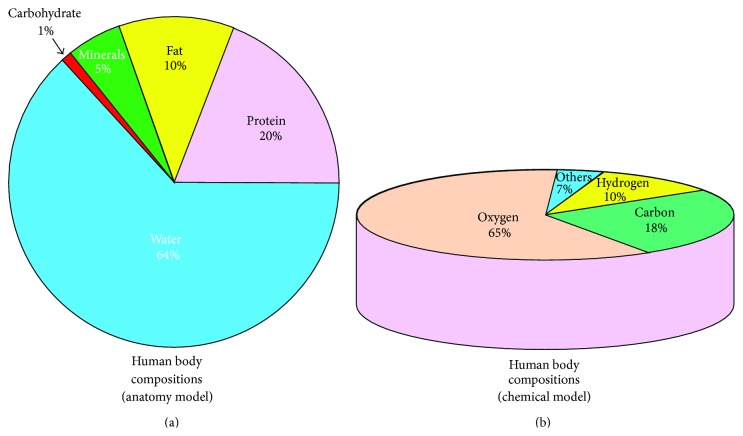
Compositions of human body: (a) anatomy model of the human body composition percentage of fat, protein, minerals, and liquid in an adult human subject; (b) chemical model of the human body composition percentage of different elements in the body composition of human subjects.

Human body is developed with the complex arrangement of different body tissues consists of water, protein, fat, and minerals called the body compositions. The human body is composed of 64% water, 20% protein, 10% fat, 1% carbohydrate, and 5% minerals approximately [15] as shown in Figure 3(a). Over 90% of the mass of the human body is made by the just three elements, namely, oxygen (65%), carbon (18%), and hydrogen (10%) among 118 elements discovered so far (Figure 3(b)). The human body compositions are largely divided into two groups: body fat and lean body. The lean body is divided to protein, mineral, and body water. Protein is a main element of muscles, whereas the minerals are found mostly in bones [43]. Total body water (TBW) consists of intracellular water (ICW) and extracellular water (ECW). ICW are found within the cells and tissues and give cell volume and tissue volumes whereas the ECW is composed of blood, lymph, and so forth [43]. Body cell mass is the sum of ICW and metabolically active tissues [43]. The body-composing constituents and their explanation and function are presented in Table 1. Body composition (Figure 3(a)) of human subject is essentially to be estimated to study and diagnose the normal health, diseases, and the other abnormal health status. Human body mass is developed with several elements (Figure 3(b)) in form of water, cells, and tissues. Human body mass consists of tissues with high conductivity called lean body mass (LBM) and tissues with low conductivity called body fat (BF). Generally, an unhealthy body composition refers to carrying too much fat in comparison to lean tissue (muscle), and the more the body's fat, the more the health risks. In fact, unhealthy body composition often leads to obesity creating many other health complications like heart disease, stroke, high blood pressure, high cholesterol, type 2 diabetes, back pain, and so forth.

### 3.5. Optimal Ratio of Body Composition for Male and Female

**Figure 4:**
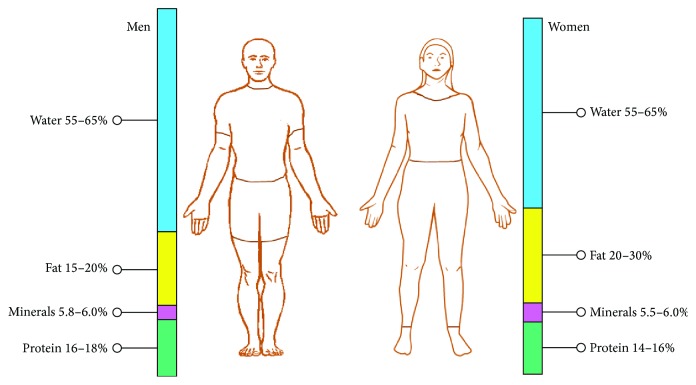
Optimal ratio of body composition.

The optimal ratio of body composition is shown in Figure 4. In human body the following relations are held for body compositions:

(1) lean body mass ∝ soft lean mass ∝ body water;
(2) lean body mass ∝ 1/percent of body fat;
(3) lean body mass varies directly as soft lean mass or body water but inversely as body fat mass;
(4) because of weight = lean body mass + body fat.

### 3.6. BIA and Body Composition Calculation

#### 3.6.1. Fat-Free Mass (FFM) or Lean Body Mass (LBM)

**Figure 5:**
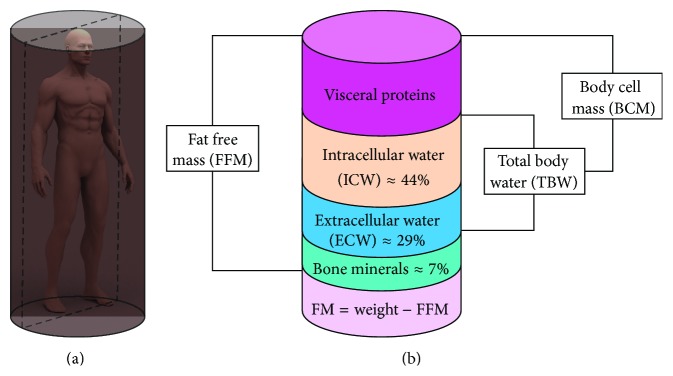
Human body as a conducting cylinder and its body composition: (a) human body assumed as a conducting cylinder in BIA, (b) body composition schematic diagram of fat-free mass (FFM), total body water (TBW), intracellular water (ICW), extracellular water (ECW), and body cell mass (BCM) [9].

The fat free mass (FFM) or the lean body mass (LBM) or muscle mass or body cell mass (BCM) [9] is developed with muscle as well as the metabolically active tissue in your organs [9]. Fat-free mass is comprised of the nonfat components of the human body. Skeletal muscle, bone, and water are all examples of fat-free mass. Several equations are proposed to calculate the fat-free mass. The schematic diagram of the body composition such as fat-free mass (FFM), total body water (TBW), intracellular water (ICW), extracellular water (ECW), and body cell mass (BCM) is shown in the Figure 5.

The following equations have low standard errors for predicting FFM and is appropriate for the general population [44]:

 for females:
  (2) Fat-Free Mass (kg) =0.475×[(ht(cm))2R(ohms)]+0.295×wt  (kg)+5.49;
 for males:
  (3) Fat-Free Mass (kg) =0.485×[(ht(cm))2R(ohms)]+0.338×wt  (kg)+3.52.

#### 3.6.2. Body Fat (BF)

The body fat is the amount of fat tissue within a body that is the total adipose tissues without any muscle tissue or body fluids or electrolyte [9]. It is the total body mass minus the FFM:

(4) Body  Fat  (BF)(kg)=Total  Body  Mass  (TBM)(kg) −Fat-Free  Mass  (kg).

#### 3.6.3. Total Body Water Body (TBW)

Several formula of total body water body (TBW) has been proposed by different research groups. Body water consists of intracellular water (ICW) and extracellular water (ECW) [18]. Total body water body (TBW) is given by [18]:

(5a) TBW  (kg)=1.84  +0.45(height2squareresistance) +0.11(weight).

According to Lukaski et al. [8] and the Jawon Medical Inc. [43], South Korea, the formula is given by:

(5b) TBW  (kg)=0.377(height2resistance)+  0.14Weight −0.08Age  +2.9Gender+4.65,

(5c) TBW  (kg)=A×(height2resistance)+B×Weight −C×Age+D×Gender,

where A, B, C, D, and E are the constants.

### 3.7. Body Tissue Conductivity

**Figure 6:**
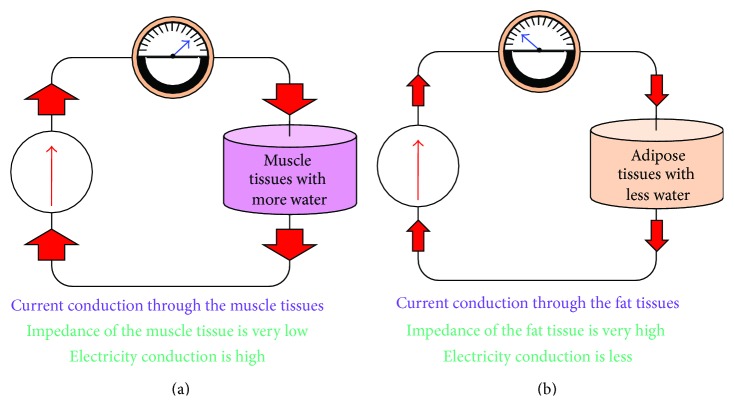
Current conduction through body tissues (a) through muscle tissue and (b) through fat tissue.

Fat tissues are the adipose tissues consisting of fat cells, which are a unique type of cell containing electrically low conductive materials. The fat tissue impedance is high as the conductivity of the fat tissue is low (Figure 6(a)). On the contrary, lean tissues contain intracellular and extracellular fluid and electrolytes. Therefore, as the conductivity of the lean tissue is high, the impedance is low in lean tissues (Figure 6(b)). Impedance is thus proportional to TBW. Under an alternating current inject to the body, the current signal flows along with paths contain more water within the body since it has high conductivity. Depending on the water content in the body, the impedance value changes for body water, body fat, and body muscle. The ratio of these two types of tissues is reflected on the electrical characteristic and the impedance value [45].

### 3.8. BIA for Body Composition and Health: The Necessity

In a healthy human body, a certain ratio of the compositions is maintained to sustain a good health. Hence, the body composition assessment is essential to study the human health as well as to diagnose a number of diseases like obesity, edema, and protein-deficient malnutrition as well as to assess the metabolic status in human body. Though there are several methods to assess the body compositions like skin-fold test [21, 45], underwater weighing [21, 45], waist-to-hip ratio method [46], and dual energy X-ray absorptiometry (DEXA) [47]; still the accurate, noninvasive, low cost, fast and easy methods are always desired in medical and clinical fields for body composition assessments. In this direction, bioelectrical impedance analysis (BIA) which is able to properly identify a subject's health risk of excessively high or low body fatness is being studied and researched by a number of research groups including doctors, clinicians, medical practitioners, and biomedical engineers. It is a growing and promising technique that ranks similar to skin-fold measurement in its accuracy, precision, and objectivity [48]. BIA is used to study, analyze, and evaluate the body fat and body fat free mass. BIA also shows many other health parameters such as your BMI (body mass index) and total body water (TBW).

### 3.9. Advantages of BIA

Compared to the other noninvasive procedures to assess the body compositions, the BIA technique has the following advantages:

- noninvasive, safe, fast, low cost, portable, easy to conduct, hazards free, and safe technique;
- measures fat-free mass and calculates fat mass;
- calculates of body cell mass, total body water, intracellular water, and extracellular water;
- BIA devices are light in weight, portable and can be used at the bedside;
- BIA devices can be used to assess total body water in individuals with altered metabolic function;
- excellent consistency for repeated measurements.

### 3.10. BIA Procedures

**Figure 7:**
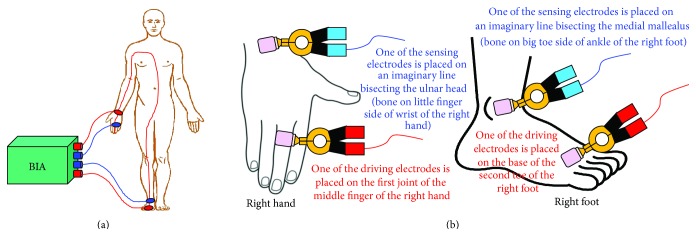
Body composition assessment using BIA: (a) BIA current injection and voltage measurement schematic; (b) electrode connection in impedance measurement procedure of the BIA.

BIA is studied with an instrument, called body composition analyzer (BCA) (Figure 7(a)), which is dedicated to inject a constant current and measure the electrical impedance of the body (*Z*_*B*_) using standard four-electrode method (Figure 7(b)). As explained earlier, in four-electrode method based BIA procedure, a low and constant amplitude (≤1 mA) alternating current (generally 50 kHz) is injected through two electrodes (outer electrodes) called current electrodes and the voltages are measured on other two electrodes (inner electrodes) named as voltage electrodes as shown in Figure 7(b). The resistance (R) or resistivity (*ρ*) is calculated from the impedance (*Z*_*B*_), and using the resistivity or conductivity, various body compositions are calculated as shown in Section 3.6.

### 3.11. Assumptions in BIA Calculations

**Figure 8:**
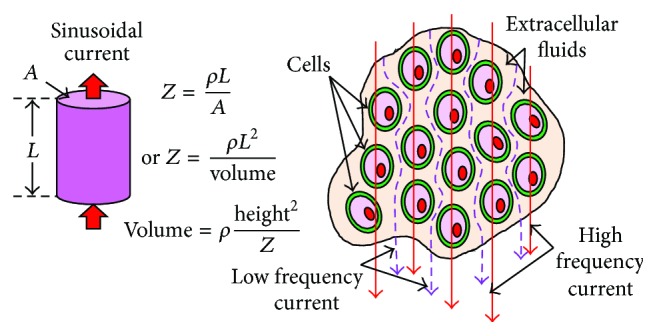
Impedance of the human body assuming it as a homogeneous cylindrical volume conductor.

The BIA assumes the human body as a cylindrical homogenous conductor (Figure 8)whose impedance is proportional to the length and inversely proportional to the cross-sectional area of the base (Figure 8).

Though, in practical case, the human body is different from the assumptions mentioned above, but for ease of calculation, the BIA formulation procedures, generally, have the following assumptions:

1. human body is a cylinder determined by height and weight;
2. body composition is homogenous and evenly distributed;
3. there are no individual differences and variations within the body compositions;
4. there are no changes in the environment (temperature), body heat, and stress.

The BIA procedure applies an alternating current which experiences a bioelectrical impedance exerted by the cells and extracellular fluids. As the cell membranes are capacitive in nature, the capacitive reactance produced by the electric current applied selectively allows the current pass through it depending on the signal frequency and hence the current paths (Figure 8). The low frequency current passes through the extracellular fluids as the cell membrane reactance does not allow the low frequency current to pass through it whereas the high frequency current penetrates the cell membranes and passes through both the extracellular fluids and the cells (membranes and intracellular fluids). Thus by applying the alternating current at a particular frequency, BIA procedure can assess the amount of extracellular water (ECW), intracellular water (ICW), and total body water (TBW = ECW + ICW).

### 3.12. BIA Outcomes

The BIA results reveal several information about the body composition and the body health such as

- LBM and BF ratio: how much lean body mass (LBM) versus body fat (BF) a person has;
- Toxic burden: how much the toxic burden of the human subject is;
- Water intake: whether the person is drinking adequate amount of water or not;
- Burning calories: how many calories the person is burning at rest;
- Loosing and gaining of LBM or BF: whether the subject is losing or gaining muscle mass or fat;
- Health status: how healthy the person's cells are.

### 3.13. BIA Preparations and Precautions

The BIA procedure is conducted on the patients by attaching few electrodes on their body and the entire experimentation is completed within 3-4 minutes. The BIA experimentation is conducted on the patients, either in lying or standing condition, with the electrodes attached to the body, and a small amount of alternation current is injected, and the potentials are measured. The amplitude of the electrical current is so small that it cannot be felt at all. The BIA procedures are conducted on the subject who should not

1. eat or drink caffeine for 4 hours prior to your appointment,
2. exercise for 12 hours prior to the person's appointment,
3. wear tight clothing or pantyhose,
4. apply lotion on the person's hands and feet prior to your appointment and
5. let the person take his diuretic medication within 6 hours prior to your appointment.

The BIA procedures are conducted on the subject who should

1. remove all metal jewellery prior to doing the assessment and
2. drink 2–4 glasses of water within 2 hours of your appointment.

### 3.14. BIA Procedure Cost

Bioelectrical impedance analysis (BIA) is a simple, safe, noninvasive, and painless procedure which is conducted by a single person: a doctor, medical practitioner, clinician, nurse, or even a laboratory expert. The approximate cost of a BIA experiment is around $35–$65 [49]. The BIA test fee in Healthy Directions, Nutrition Therapy, and Counseling Center of Doylestown Hospital, USA, is $25 [50].

### 3.15. Single Frequency BIA and Dual Frequency BIA

Researches show that the alternating current BIA can be performed in either single frequency current (called single frequency BIA or SFBIA) or dual frequency current (called dual frequency BIA or DFBIA) [51]. Generally, SFBIA is performed with 50 kHz and DFBIA is performed with 50 kHz and 200 kHz. TBW can be measured using SFBIA and TBW and ECW can be calculated by DFBIA. By measuring the impedance (*Z*_*B*_) at 50 kHz and 200 kHz and by applying predictive equations (5a), (5b), (5c), and (6), both extracellular water (ECW) and TBW, respectively, are calculated and the intracellular water (ICW) is deduced [45]. The extra-cellular mass (ECM) and body cell mass (BCM) are found from ECW and ICW, respectively [45]. Several equations are proposed and used to predict fat-free mass (FFM). ECW can be found related to extracellular mass (ECM) and ICW to body cell mass (BCM). The equation for SFBIA and DFBIA is shown in (5a), (5b), (5c), and (6):

(6) TBW(Liters)=[(0.3963×height2)impedance(50 kHz)] +(0.143×weight)+8.4,

(7a) ECW(Liters)=[(0.178458×height2)impedance(5 kHz)] +(0.06895×weight)+3.794,

(7b) TBW(Liters)=[(0.24517×height2)impedance(200 kHz)] +(0.18782×weight)+8.197.

## 4. Electrical Impedance Spectroscopy (EIS)

**Figure 9:**
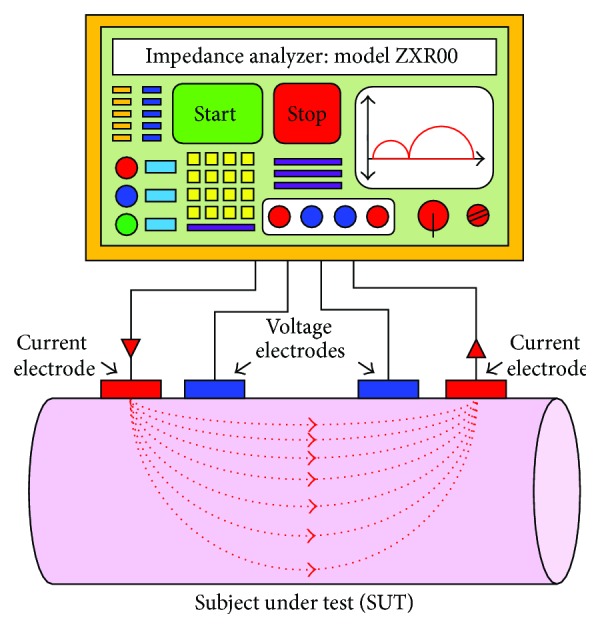
Electrical impedance spectroscopy (EIS) studies on biological materials with four-electrode technique using an impedance analyzer.

Electrical impedance spectroscopy (EIS) [52–54] estimates the complex electrical impedance (*Z*(*f*)) and its phase angle (*θ*(*f*)) of a subject under test (SUT) at different frequency points *f*_*i*_  (*f*_*i*_:*f*_1_, *f*_2_, *f*_3_,…, *f*_*n*_) by measuring the surface potentials (*V*(*f*)) developed for a constant current injection (*I*(*f*)) at the boundary through a linear array of the surface electrodes attached to the SUT (Figure 9). In EIS, a frequency dependent constant amplitude sinusoidal current (*I*(*f*)) is injected through either two-electrode method or four-electrode method. As explained earlier, in 2-electrode based EIS and 4-electrode based EIS methods, the surface electrodes through which the current signal is injected are known as the current electrodes or the driving electrodes (as shown by the red colored electrodes in Figure 9) and the electrodes on which the frequency dependent ac potential (*V*(*f*_*i*_)) is measured are called voltage electrodes or sensing electrodes (as shown by blue colored electrodes in Figure 9). Therefore, in EIS the frequency dependent electrical bioimpedance *Z*(*f*_*i*_) is found as the transfer function of the SUT, and thus the *Z*(*f*_*i*_) is calculated by dividing the voltage data (*V*(*f*_*i*_)) measurement by applied current (*I*(*f*_*i*_)) as shown in

(8) $$\begin{array}{c} Z\left(f_{i}\right)=\frac{V\left(f_{i}\right)}{I\left(f_{i}\right)}. \end{array}$$

EIS can be potentially used as a nondestructive evaluation technique [55] for a number of applications in the field of science engineering and technology. The EIS-based frequency response studies on the electrical impedance of any material can provide its structural and compositional properties as well the frequency response of the material properties which can be potentially used for nondestructive material characterization [52–54]. Therefore the EIS has been suitably applied in several fields such as electrochemistry and chemical engineering [54, 56–61], material engineering [62–71], biomedical engineering [72–76], civil engineering [77–79], wood science [80, 81], plant physiology [82–84], microfluidics [85, 86], material engineering [87–90], fuel cell technology [91], and MEMS and thin films [92, 93] and so on.

## 5. Impedance Plethysmography (IPG)

Impedance plethysmography (IPG) is a electrical impedance based noninvasive medical diagnostic procedure which measures small changes in the blood volume in terms of its electrical bioimpedance of a body part, say chest, calf, or other regions of the body, to study the tissue health condition of the patient. The impedance measured in IPG provides the information about the tissue health of the body tissue, and hence it can be suitably used to indirectly study and analyze the tissue health and function of a patient under test. Therefore, the IPG can be considered as a bioimpedance analyzing technique that measures the change in blood volume for a specific body segment by measuring the electrical impedance which changes with a change in the blood volume.

### 5.1. IPG Procedure

**Figure 10:**
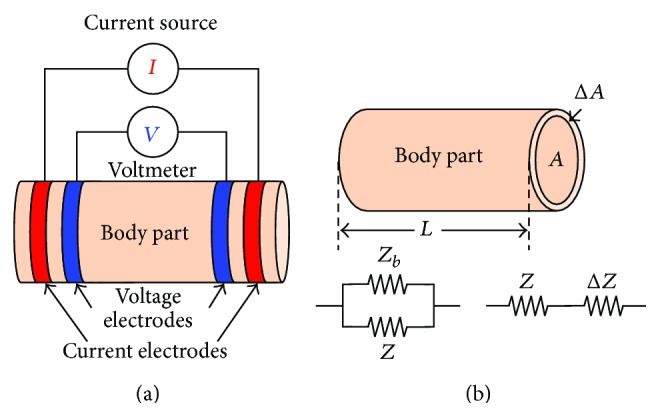
The schematic of the impedance measurement in IPG with four-electrode method and the calculation of limb volume in terms of bioimpedance: (a) impedance measurement of body part with four-electrode method, (b) volume change in body part due to blood pressure pulse and corresponding impedance variation due to the volume change.

In IPG, four conductive bands are taped around the body part or a limb and the limb impedance change due to the blood circulation is measured to calculate the blood volume changes (Figure 10(a)). A low amplitude, low frequency (50 kHz) ac current is passed through two outer electrodes and the change in electrical impedance is measured (Figure 10(b)) across the inner electrodes. In early 1950's, Nyboer [94, 95] proposed the impedance plethysmography equation to evaluate the volumetric change in the body parts in terms of impedance. But the formula proposed by Swanson and Webster, 1976 [96] is more simpler both mathematically and computationally. The formula works on three assumptions: the expansion in the artery is uniform, blood resistivity (*ρ*_*b*_) does not change, and current fluxes are parallel to the artery (Figure 10(b)). In the theory of IPG, for each pressure pulse produced due to the blood flow through limb having an artery with an volume *V* and cross sectional area *A*, an extra amount blood with a volume of Δ*V* enters to that limb by increasing the limb volume from *V* to *V* + Δ*V* and consequently a shunting impedance (*Z*_*b*_) is assumed to be produced. Therefore the (*Z*_*b*_) is given by [96, 97]

(9) $$\begin{array}{c} Z_{b}=\rho_{b}\frac{L}{\Delta A}. \end{array}$$

Hence, the artery volume change is given by [96, 97]

(10) $$\begin{array}{c} \Delta V=L\cdot \Delta A=\rho_{b}\frac{L^{2}}{Z_{b}}. \end{array}$$

For each pressure pulse, the area of the artery (*A*) increases from *A* to *A* + Δ*A* (Figure 10). Consequently, the artery impedance (*Z*_*b*_) is reduced by adding an impedance (Δ*Z*) produced by the Δ*A* and this *Z*_*b*_ will be connected in parallel to *Z* [96, 97].

As we measure the Δ*Z* rather that *Z*_*b*_, the *Z*_*b*_ is required to be replaced by Δ*Z*. Now the blood impedance change, Δ*Z*, is given by [96, 97]:

(11) $$\begin{array}{c} \Delta Z=\left(Z_{b}||Z\right)-Z=-\frac{Z^{2}}{Z+Z_{b}}. \end{array}$$

Now as, *Z*_*b*_ ≫ *Z*, the *Z*_*b*_ is given by [96, 97]:

(12) $$\begin{array}{c} \frac{1}{Z_{b}}≅-\frac{\Delta Z}{Z^{2}}. \end{array}$$

Therefore, the increases in the limb volume Δ*V* is given by [96, 97]:

(13) $$\begin{array}{c} \Delta V=-\rho_{b}\frac{L^{2}\Delta Z}{Z^{2}}. \end{array}$$

If all the assumptions are valid, then the volumetric change in a body part due to blood flow can be calculated from the impedance measurement using IPG technique.

Peterson [98] has conducted the experiments conducted on an impedance plethysmograph suitable for ambulatory blood pressure monitoring. The impedance plethysmograph, developed and used in the experiment worked as an ambulatory blood pressure monitor (ABPM), contained four circular copper band electrodes attached to the upper left forearm of a volunteer subject. In ABPM, the impedance was measured by using a signal generator connected to the outer two bands for electric current injection, and an amplifier circuit connected across the inner two electrodes for voltage measurement [98].

### 5.2. Advantages

Compared to the other techniques, the Impedance Plethysmography (IPG) has a number of advantages as listed below:

- noninvasive;
- low cost;
- fast process
- portable and easy to operate and dry type testing procedure;
- bedside measurement and ambulatory measurement are possible;
- measurement in ICU is possible;
- current density in IPG with ring electrodes is more uniform compared to other four electrode methods using spot electrodes;
- skin electrode impedance effect is less and skin impedance can be further reduced by applying the electrode gel;
- electrical signal in IPG is easily to control, process and acquire;
- IPG is less temperature dependent;
- surrounding humidity effect in IPG is less;
- applicable for healthy persons and patients of all age groups;
- IPG helps doctors and clinicians to measure changes in venous blood volume as well as the arteries pulsations;
- compared to venography, which is invasive and requires a skilled person to perform and interpret accurately, IPG is an easy to perform and understand;
- ICG helps a doctor to detect deep vein thrombosis (DVT);
- IPG is found 96% sensitive and 98% specific in the diagnosis of arterial occlusive disease and more than 85% sensitive in the diagnosis of DVT and valvular diseases.

### 5.3. Limitations

IPG has the following limitations:

- electric current is injected to the subject;
- electro skin impedance error is introduced.

### 5.4. Applications

Due to the number of advantages of the IPG, it has been used for several application as follows:

- blood volume measurement;
- detection of deep vein thrombosis (DVT);
- detection of venous and arterial insufficiency;
- screening of the patients who are likely to have blood clots in legs;
- detection of the source of blood clots in the lungs (pulmonary emboli);
- indirect assessment of central and peripheral blood flow;
- detect blood flow disorders such as venous and arterial occlusive diseases (estimate severity);
- early stage arteriosclerosis;
- functional blood flow disturbances, migranes;
- general arterial blood flow disturbances.

## 6. Impedance Cardiography (ICG)

### 6.1. Introduction

**Figure 11:**
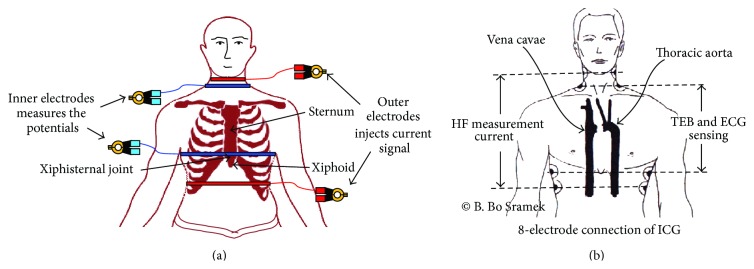
The schematic of the impedance measurement in ICG with four-electrode method: (a) impedance measurement with band electrode (photograph courtesy: Ref [100]) and (b) impedance measurement with band electrode (photograph courtesy: http://www.hemosapiens.com/teb.html (with permission of B. Bo Sramek), Retrieved on: 12.12.2013).

Impedance cardiography (ICG), also referred to as transthoracic electrical impedance plethysmography, is a technology which calculates the changes in blood volume in transthoracic region over time in terms of the changes in transthoracic impedance (Figure 11) called thoracic electrical bioimpedance (TEB) or *Z*_*o*_. Body impedance is changed in human body due to the blood circulation caused by each heart rhythms, and hence the transthoracic impedance (*Z*_*o*_) is influenced by its biological composition, breathings, and by the blood circulation and blood volume in the transthoracic region. Therefore, analyzing the *Z*_*o*_, the health of the heart along with a number of hemodynamic parameters can be evaluated. ICG has been researched since the 1940s. NASA developed the technology in the 1960s [99].

### 6.2. ICG Procedure

The ICG procedure is basically similar to the IPG procedure in which the impedance measurement is conducted by injecting a low amplitude constant alternating electric current (frequency range of 20 kHz–100 kHz) [58] into the volume conductor and measuring the corresponding voltage (Figure 11). The frequency dependent impedance *Z*(*f*) is measured from the ratio of voltage *V*(*f*) to current *I*(*f*) applied, usually the DC value is eliminated, and only the impedance variation Δ*Z* is further examined [100]. The current injection and the voltage measurement are conducted with four-electrode method either using the ring electrode or the spot electrodes (common ECG electrodes). In ring electrode based ICG system, four ring electrodes are required, whereas the ICG with spot electrode configuration needs eight ECG electrodes. Hence, all the ICG systems are operated with four-electrode measurement technique using either four-band electrodes (Figure 11(a)) or 8 spot electrodes (Figure 11(b)). The 4-electrode and 8-electrode connections of ICG are shown in Figures 11(a) and 11(b), respectively. In ring electrode connection, one electrode of the driving electrodes (the outer pair) is placed around the abdomen and the other is placed around the upper part of the neck (Figure 11(a)). For the sensing electrode pairs (inner electrode pair), one electrode is placed around the thorax at the xiphisternal joint and the other around the lower part of the neck (Figure 11(a)). In 8-electrode connections of ICG also the current injection and voltage measurement are conducted using four-electrode method as the current is injected through the two upper most electrodes and two lower most electrodes and the voltage data are collected across the inner two sets (each set contains two spot electrodes) of the electrode. In recent times the band electrodes are often replaced with spot electrodes such as normal ECG electrodes [100]. A low magnitude, low frequency ac signal, is injected by current (red in the figure) electrodes. Voltage developed across voltage electrodes due to the current injection through the current electrodes is measured and the average transthoracic impedance (*Z*_*o*_) across the transthoracic region and the small change in impedance (Δ*Z*) due to blood flow are calculated and monitored against time. After getting the *Z*_*o*_, *dZ*/*dt*, Δ*Z* are calculated. Analyzing the *Z*_*o*_, *dZ*/*dt*, Δ*Z*, the stroke volume, cardiac volume, and several hemodynamic parameters are calculated for noninvasive diagnosis of the heart and circulatory system.

**Figure 12:**
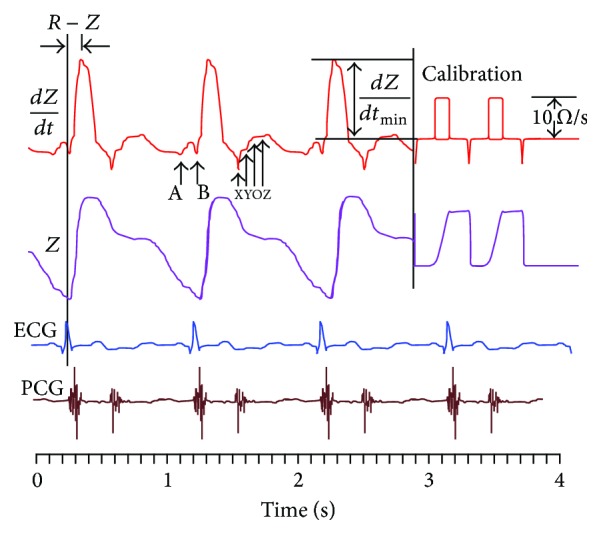
The transthoracic impedance curve [100] over time.

Figure 12 shows a typical thorax impedance curve (*Z*), its first time derivative (*dZ*/*dt*), the simultaneous electrocardiogram (ECG), and phonocardiogram (PCG) curves [100]. It is important to note that a decrease in impedance results in an increase in the *y*-axis magnitude, and hence this sign convention describes the changing transthoracic admittance. Thus a decrease in impedance can be explained by an extra amount of blood (low impedance material) flow in the thorax. It is also important to note that the polarity of the first derivative curve (*dZ*/*dt*) is consistent with the impedance (*Z*) curve.

### 6.3. ICG and Hemodynamic Parameters

In ICG, a number of hemodynamic parameters are derived to study the hemodynamic health and functions including the following:

- stoke volume;
- cardiac volume;
- stroke volume/stroke volume index (SV/SVI);
- cardiac output/cardiac index (CO/CI);
- systemic vascular resistance/index (SVR/SVRI);
- velocity index (VI);
- thoracic fluid content (TFC);
- systolic time ratio (STR);
- left ventricular ejection time (LVET);
- preejection period (PEP);
- left cardiac work/index (LCW/LCWI);
- heart rate (HR).

#### 6.3.1. Hemodynamic Parameter Calculation

The following section represents the detail explanations and the formula of the hemodynamic parameters [100, 101].

*Stroke Volume*. Stroke volume is very important in cardiovascular physiology to determine the cardiac function and cardiac health and cardiac parameters such as cardiac output (CO), ejection fraction (CEF) and so forth Stroke volume is measured in milliliters per beat (mL/beat) which is almost equal for each ventricles (approximately 70 mL/beat for a normal adult subject). SV is indexed to a patient's body size by dividing by body surface area (BSA) to yield stroke index (SI). Using the information acquired from the echocardiography for a given ventricle, the SV is estimated by subtracting volume of the blood in the ventricle at the end of a beat (called end-systolic volume or ESV) from the volume of blood just prior to the beat (end-diastolic volume or EDV). Using the impedance method, the SV is calculated by the formula as described below.

(i) stroke volume = [*ρ*] × [*L*/*Z*_*o*_]^2^ × Δ*Z* (Nyboer Formulae);
(ii) stroke volume = [*ρ*] × [*L*/*Z*_*o*_]^2^ × *dZ*/*dt* × LVET (Kubicek Formulae0);
(iii) stroke volume = [*V*/*Z*_*o*_] × *dZ*/*dt* × LVET (Sramek Formulae),

where *ρ* is specific resistivity of blood which is a constant for a particular subject, *L* is transthoracic length, *Z*_*o*_ is average transthoracic impedance, LVET is left ventricular ejection time, *V* is volume of the transthoracic tissues involved electrically during the test = *L* ^3^/4.25.

*Cardiac Volume*. The cardiac volume refers to the volume of the heart which is usually relating to the volume of blood contained within it at various periods of the hear cycle.

*Cardiac Output (CO)*. Cardiac output (CO) is the total amount of blood ejected or pumped out by the left ventricle of the heart into the systemic circulation in one minute. Hence the CO is found as equal to the stroke volume times the heart rate. Using the cardiac output of a human subjects, one more hemodynamic parameter, called the cardiac index (CI), is calculated by dividing the CO by the body surface area (BSA). The CO is measured in (L/min or mL/min) and as the CI is measured in litres per minute per square metre (L/min/m^2^). The CO is, generally, found as approximately 5.6 L/min for a human male subject and 4.9 L/min for a human female subject. Thus the CO and the CI are found as:

 CO = (stroke volume × heart rate);
 CI = cardiac output/body surface area.

### 6.4. Advantages

The impedance cardiography (ICG) has a number of following advantages:

- noninvasive, low cost, fast, portable, safe;
- current distribution is better; and less noise effect;
- electrode-skin impedance effect being less.

## 7. Electrical Impedance Tomography (EIT)

### 7.1. Introduction to EIT

**Figure 13:**
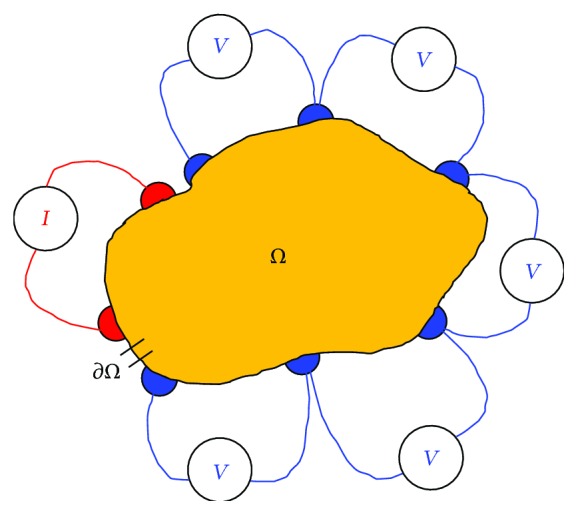
A closed domain of interest under EIT scanning with a constant current injection through driving electrodes and boundary potential measurement on sensing electrodes.

Electrical impedance tomography (EIT) [102–114], a computed tomographic image reconstruction technique, is a nonlinear inverse problem in which the electrical conductivity or resistivity of a conducting domain (Ω) is reconstructed from the surface potentials developed by a constant current signal injected (Figure 13) at the domain boundary (∂Ω). A low frequency, constant amplitude sinusoidal current is injected to the boundary (∂Ω) of the object domain (Ω) to be imaged within a volume conductor surrounded by an array of surface electrodes and the boundary potentials are measured using an electronic instrumentation [115–123]. Constant current signal is injected to the closed domain under test (DUT) through the different pairs of electrodes called driving electrodes and the voltage data are collected from the other electrodes or electrode pairs called sensing electrodes, and the set of voltage current data obtained from this noninvasive boundary measurements is used to reconstruct the conductivity distribution of the DUT. Boundary data are then sent to the PC and the data are processed in PC to reconstruct the spatial distribution of the electrical conductivity of the DUT using a computer program called image reconstruction algorithm [124–133].

EIT is a low cost, portable, fast, noninvasive, nonionizing, and radiation free technique, and hence it is found advantageous in several fields of applications application compared to the other computed tomographic methods like X-ray CT [134–136], X-ray mammography [137], MRI [138, 139], SPECT [140, 141], PET [142, 143], ultrasound [144, 145], and so forth. EIT has been applied in a number of research areas such as medical imaging clinical diagnosis [146–152], chemical engineering [153], industrial process application [154, 155], material engineering [156], microbiology and biotechnology [157, 158], nondestructive testing (NDT) in manufacturing technology [159], civil engineering [160], earth science and geophysics and geoscience [161], defense fields [162], archaeology [163], oceanography [164], environmental engineering [165], and other fields of applied science, engineering and technologies [166].

### 7.2. Physics of EIT

**Figure 14:**
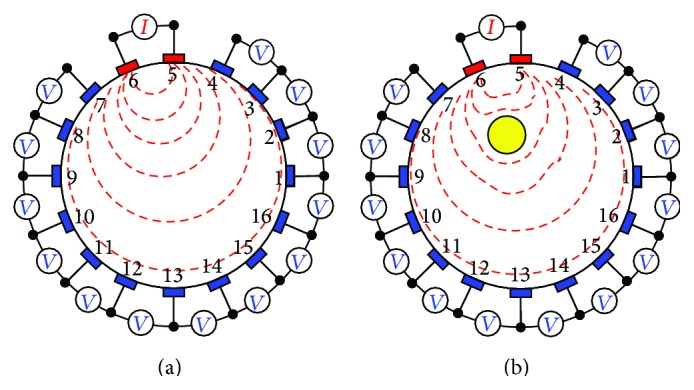
Variation in boundary data profiles for a homogeneous domain and a inhomogeneous domain (a domain with inhomogeneity) due to the variation in the profiles of impedance distributions (a) domain without inhomogeneity, (b) domain with inhomogeneity.

In EIT, when a constant current is injected into DUT, the current signal conducted through the domain produces current fluxes which induce potentials within the DUT. The potential profiles developed by the current conduction depend on the profiles of the current fluxes which are influenced by the impedance profile of the DUT. As the profile of the current conduction in a homogeneous domain (Figure 14(a)) differs from the current flux lines produced by the inhomogeneous DUT (domain with inhomogeneity) (Figure 14(b)), the voltage profile of the homogenous DUT will be different from the domain with inhomogeneity. Similarly, the boundary potential profiles will depend on the domain impedance distribution, and hence the information of the impedance distribution of the DUT is hidden inside the boundary potential data.

### 7.3. Comparison with CT

EIT is a computed tomographic imaging modality which uses an electrical energy (either by injecting electrical current or voltage signals) and the developed potentials are collected at the domain boundary, whereas in X-ray CT, X-ray beams are passed through the SUT at different angles, called the projection angles, and the attenuated X-ray beams are collected by the X-ray detectors. The image reconstruction algorithm uses the measured data in PC and reconstructs the spatial distribution of the electrical impedance and the X-ray attenuation coefficient of the SUT in EIT and CT, respectively. Therefore, the CT is a tomographic technique imaging with X-rays and EIT reconstructs the tomographic images with electric current.

### 7.4. Advantages of EIT

EIT has several advantages over other computed tomographic imaging techniques used for medical imaging applications as summarized below:

- noninvasive;
- radiation free;
- nonionizing method;
- fast data acquisition;
- high temporal resolution;
- medically safe process;
- low cost device;
- portable device and easy to use;
- suitable for bedside measurement and ICU monitoring;
- suitable for ambulatory monitoring;
- negligible patient preparation is required;
- no precautions are required;
- no postexperimental discomfort;
- no postexperimental precautions and restrictions;
- suitable for the patients of any age groups as well as critically ill patients.

### 7.5. Capacitance Tomography and Resistance Tomography

In EIT the spatial distribution of the electrical conductivity is reconstructed from the boundary data collected by an alternating current injection at the domain boundary. In some applications the electrical permittivity of the DUT is reconstructed from the voltage current data collected at the boundary and the imaging modality is called electrical capacitance tomography (ECT) [125, 133, 167, 168] which is generally used in industrial process application and mechanical and material engineering. The electrical resistivity of the domain under test is sometimes reconstructed from the boundary data generated by a direct current injection in industrial process imaging, civil structure imaging, subsurface imaging, and other applications of geotechnology. This method is known as the electrical resistance tomography (ERT) [169–173].

### 7.6. Difference EIT and Static EIT

EIT image reconstructions are conducted from the boundary data either with the difference or dynamic imaging modality called the difference imaging or another modality called static imaging. In difference imaging, the impedance distribution of the DUT is reconstructed by comparing the data collected from the domain with inhomogeneity and the data collected from the homogeneous domain (reference data), whereas the static imaging reconstructs the conductivity distribution from the data collected from the inhomogeneous medium without using the reference data set. The present EIT technologies are generally found applied in difference imaging modalities as the static imaging seems to be difficult and more influenced by the measurement errors, and hence the static EIT is found still far from clinical applications primarily. Though the static EIT is found difficult due to the fundamental ill-posedness of the EIT inverse problem, but still it is little bit too early to say that the static EIT is not to be pursued since active researchers are still looking for innovative algorithms and new measurement technologies.

The dynamic or difference EIT that was introduced by Barber and Brown in 1984 [174] produces differential images, whereas the static imaging yields absolute images. The difference impedance imaging may be either the time difference EIT [105, 175, 176] or frequency difference EIT [105, 177, 178]. The time difference EIT imaging produces the images of the variation of the conductivity distribution of a region between two different time intervals [104, 179], whereas the frequency difference imaging reconstructs the impedance distribution from two data sets collected at two discrete frequencies. The difference EIT allows us to monitor the changes such as gastric emptying or long-term observation of body functions/volume changes [104, 179] including the visualization of physiological activities in a human body such as respiration. It can also be used for breast cancer detection, cardiac circulation, brain function, stomach emptying, fracture healing, bladder filling, and others. Difference EIT can also be used in nonmedical applications such as in corrosion detection, crack detection, electric field sensing, bubble detection, and other nondestructive testing.

### 7.7. Multifrequency EIT System

As the different biological tissues have different frequency versus impedance responses [5, 76, 180, 181], the multifrequency EIT [182–185] is found more effective and efficient in biological tissue characterization, medical imaging, and clinical diagnosis of the human diseases. The multifrequency EIT provides a lot of information about the tissue health which can be potentially utilized for better tissue characterization. Moreover, the multifrequency EIT can provide all the parameters in the frequency domain which adds another advancement in the diagnosis and treatment of the disease. Thus, the multifrequency impedance imaging can be suitably applied in diagnosis and treatment of the human diseases, tumors, lesions, and cancers.

### 7.8. 3D EIT

2D electrical impedance tomography (2D-EIT) reconstructs the approximate spatial distribution of the internal impedance profile of a DUT from the boundary measurements of voltage-current data developed by a constant current injection through the surface electrodes fixed in a 2D plane within the patient's body [104]. Thus, the 2D EIT approximately reconstructs the 2D conductivity distribution by assuming the electrical current conducts in a 2D plane. But as the electrical current is not confined in the plane of electrode array rather it spreads over a 3D space within the volume conductor, the 2D EIT suffers from the errors contributed by this 3D conduction of electrical current [104]. Moreover, 2D EIT of a tissue under test provides only the 2D impedance distribution in its tomograms in which the 3D anatomical and physiological information is not available. But, 3D EIT provides 3D conductivity distribution with a better and more scientific visualization of the tissue interiors which helps the doctors and clinicians with better tissue characterization [104].

### 7.9. A Basic EIT System

**Figure 15:**
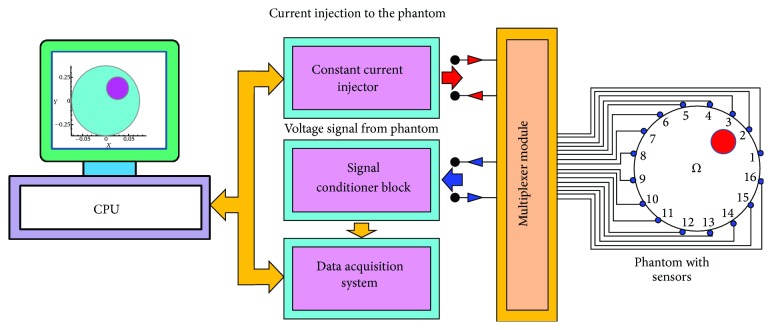
Schematic of a basic EIT system with patients with surface electrodes attached to its transthoracic region for thoracic imaging.

A basic EIT system contains three parts (Figure 15): EIT instrumentation [115–123], a PC with reconstruction algorithm [124–133], and an array of EIT sensors or surface electrodes [57, 102, 106, 110, 186] attached to a practical phantom or a subject under test (SUT).

In EIT, an array of sensors or surface electrodes [57, 102, 106, 110, 186] is attached to the boundary of the DUT. A low frequency sinusoidal current of constant amplitude is generated by a constant current source and it is injected through the driving electrodes and the boundary potentials are measured on the sensing electrodes using a particular current injection and voltage measurement fashion called the current pattern [100, 187, 188]. Boundary potentials are measured using a data acquisition system (DAS) [108, 121, 123] and the processed data obtained from the data processing circuit containing amplifier and filters (Figure 15) are sent to the PC for computation and image reconstruction. Thus a basic EIT system has three main parts:

- EIT-instrumentation;
- PC with reconstruction algorithm;
- electrode array or EIT sensors attached to the subject under test (SUT).

### 7.10. EIT Instrumentation

A common EIT Instrumentation has four main parts:

- constant current injector (CCI);
- signal conditioner block (SCB);
- electrode switching module (ESM);
- data acquisition system (DAS).

Constant current injector (CCI) may be developed with a signal/function generator and a voltage controlled current source (VCCS) which is used to inject a constant current signal to the domain boundary through the driving electrodes. The developed voltage signals on the sensing electrodes are processed by signal conditioner block (SCB) and sent to the DAS for data acquisition. The boundary data are collected with a particular current pattern by switching driving and sensing electrode in a particular fashion using a electrode switching module (ESM). The boundary data collected from a complete EIT scan of the object are sent to the PC for image reconstruction.

### 7.11. Electrode Array or EIT Sensors

Surface electrode array is a very important part of an EIT system as the boundary data quality, and thus the image quality depends on it. As the EIT electrodes are used to inject current to the body under test and collecting the voltage data at boundary electrodes, electrode parameters are very crucial in EIT imaging. For designing a better EIT electrode system, one should carefully select the electrode parameter such as electrode number, electrode material, electrode geometry (shape and size), electrode model (point, gap, shunt, and complete) or electrode type (noncompound or compound).

### 7.12. Reconstruction Algorithm

Image reconstruction algorithm is one of the most important parts of EIT system as the image reconstruction is conducted by the algorithm. The boundary potential data collected from the real objects (SUT) are sent to the PC and are processed to reconstruct the EIT images using the EIT reconstruction algorithm. EIT image reconstruction algorithm has generally two main parts:

- forward solver (FS);
- inverse solver (IS).

The forward solver solves the characteristic equation or governing equation of EIT [102–110, 189–194] and computes the boundary potentials called calculated boundary data (*V*_*c*_) for a known constant current simulation [*C*] in PC. The calculated potential data (*V*_*c*_) are then compared with the measured potential data (*V*_*m*_) in inverse solver (IS) and the conductivity distribution of DUT is reconstructed for which the difference between *V*_*m*_ and *V*_*c*_  (Δ*V* = *V*_*m*_ − *V*_*c*_) is minimized.

The Gauss-Newton based EIT image reconstruction algorithm (GN-EIRA) is generally developed with a finite element method (FEM) [189–196] based forward solver (FEM-FS) a Gauss-Newton based inverse solvers (GN-IS) in MATLAB. The GN-EIRA is developed with Gauss-Newton based minimization algorithm (GNMA) and Newton Raphson iterative technique (NRIT) [189–196]. The forward solver generally applies a numerical technique like FEM or boundary element method (BEM) [196] or else and calculates the nodal potentials [195] within the discretized domain. Using the forward solution data, the algorithm construct the sensitivity matrix, called Jacobean (*J*) which is used by the inverse solver. The inverse solver uses the voltage difference vector (Δ*V*) and *J* and calculates a correction or update in conductivity distribution to modify it for minimizing the voltage data mismatch. Inverse solver repetitively solves the forward solution and tries to compute an approximate conductivity distribution for which the difference between measured and calculated voltage data is minimized.

#### 7.12.1. GN-EIRA

**Figure 16:**
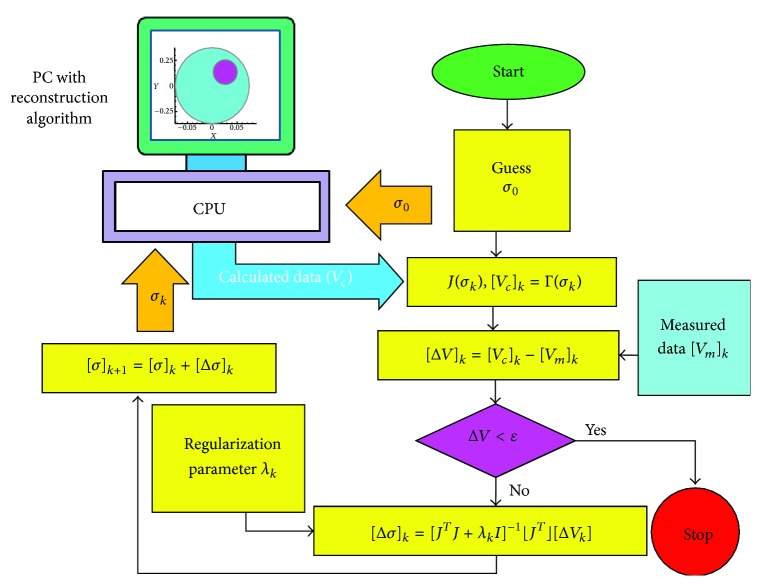
The flow chart of a standard EIT imaging reconstruction algorithm.

In EIT, the GN-EIRA is developed with a FEM based forward solver (FS) and Gauss-Newton method based inverse solver (GNIS), whereas the GNIS is developed with Gauss-Newton based minimization algorithm (GNMA) and Newton Raphson Iterative technique (NRIT). The forward solver solves the EIT governing equation by developing the forward model of the EIT problem and calculates the boundary potentials (*V*_*c*_) for a known current injection [*C*] applied to the boundary of the domain with a known conductivity distribution [*σ*_0_]. The solution obtained from the FP is also used to compute the potential mismatch vector [Δ*V* = *V*_*m*_ − *V*_*c*_] and the Jacobian (*J*) that are used in inverse problem to compute the conductivity update vector [Δ*σ*]. The GNIS solves the inverse problem (IP) using the boundary data [*V*_*c*_] computed by the forward solver and tries to estimate the domain conductivity distribution from measured boundary potential data (*V*_*m*_) developed for the real current injection of the same amplitude. The GNIS in EIT repetitively calculates the conductivity update [Δ*σ*] vector and simultaneously reconstructs the conductivity distribution [*σ*] in each iteration by updating the conductivity distribution obtained in previous iteration. The iteration process is continued to update the conductivity distribution with modified NRIT until a specified error limit criterion (*ε* = *f*(Δ*V*)) is obtained (Figure 16).

#### 7.12.2. Governing Equation of EIT

The EIT reconstructs the conductivity of a closed domain from the boundary voltage current data using the relationship of the domain conductivity distribution and the domain potentials distribution which is called the EIT governing equation. In EIT, the conductivity distribution of a DUT could be defined with the distinct values of electrical conductivities at each points within the domain which are associated with their corresponding coordinates. Now, if an electric current of constant amplitudes is injected to the domain boundary (∂Ω), the current flux will develop a particular potential profile within DUT. The developed potential profile will be the function of the current amplitude and the conductivity profile of the domain. Therefore, the “EIT governing equation” could be obtained as the relationship between the elemental electrical conductivity (*σ*_*e*_) of the domain and their corresponding potential values (Φ) using the Maxwell's equations. EIT governing equation can be represented as a partial differential equitation [102–110, 189–195] as shown in

(14) $$\begin{array}{c} \nabla \cdot \sigma \nabla \phi =0. \end{array}$$

#### 7.12.3. Boundary Conditions

The forward solver of EIT reconstruction algorithm discretizes the DUT with a finite element mesh containing a finite number of triangular elements and the finite number of nodes and applies the FEM formulation technique on the EIT governing equation (14) of the DUT. As the EIT governing equation is a nonlinear partial differential equation, it has an infinite number of solutions, and hence, to restrict its solutions space, the FEM based EIT forward solution process essentially needs the boundary conditions [1–5] which provide some specified value of certain system parameters which may be either the potentials at the surface or the current density crossing the boundary or mixed conditions.

The boundary conditions, in which the parameters are the potential at the domain boundary (∂Ω), are named as the Dirichlet boundary conditions which are represented as [102–110, 189–196]

(15) $$\begin{array}{c} \Phi =\Phi_{i}, \end{array}$$

where *i* = 1,…, *d* are the potentials on the nodes under electrodes (*d* is number of electrodes potentials).

On the other hand, the boundary conditions of EIT which specify the current density crossing the boundary (∂Ω) are known as the Neumann boundary conditions [102–110, 189–196] defined as:

(16) $$\begin{array}{c} \overset{}{\underset{\partial \Omega }{\int }}\sigma \frac{\partial \Phi }{\partial n}=\{\begin{array}{cc} +I & \mathrm{on}\;\text{the}\;\text{source}\;\text{electrode}\\ & \left(I\;\text{is}\;\text{the}\;\text{injected}\;\text{current}\;\text{amplitude}\right),\\ -I & \mathrm{on}\;\text{the}\;\text{sink}\;\text{electrode}\\ & \left(I\;\text{is}\;\text{the}\;\text{injected}\;\text{current}\;\text{amplitude}\right),\\ 0 & \text{otherwise}. \end{array} \end{array}$$

#### 7.12.4. Forward Solution

In EIT, the FS derives the forward model of a DUT from its governing equation by applying the FEM formulation technique on (1) and imposing the boundary conditions. Using the initial guessed conductivity distribution [*σ*_0_] and nodal coordinates, the FS develops the forward model which is a system of equations represented in the form of a matrix equation (17). The forward model, in form of the matrix equation, actually represents the relationship between the current injection matrix [*C*] (matrix of the applied current signal) and the nodal potential matrix [Φ] (matrix of the developed nodal potential data) through the transformation matrix [*K*(*σ*_*e*_)] as given below [102–110, 189–195]:

(17) $$\begin{array}{c} \left[\Phi \right]={\left[K\left(\sigma_{e}\right)\right]}^{-1}\left[C\right], \end{array}$$

where *σ*_*e*_ is the elemental conductivity distributions.

The FS in EIT thus develops the forward model of the DUT and calculates the boundary potential (*V*_*c*_) data for a known conductivity distribution (initial guess), [*σ*_*e*_ = *σ*_0_], and a known simulated current injection [*C*] (available from the boundary conditions).

#### 7.12.5. Inverse Solution

If *F* is a function mapping an e dimensional (e is the number of element in the FEM mesh) impedance distribution into a set of *d* (*d* is the number of the experimental measurement data ([*V*_*m*_]) available) approximate measured voltages, then the conductivity of the DUT can be estimated from the boundary data by applying the Gauss-Newton method based minimization algorithm [102–110, 189–195] which tries to find a least square solution of the minimized object function *s*_*r*_ [102–110, 189–195] which is defined as

(18) sr=12||Vm−F||2+12λ||Rσ||=12(Vm−F)T(Vm−F)+12λ(Rσ)T(Rσ)2,

where *F* represents the forward model predicted boundary potential data or *V*_*c*_, *R* is a matrix which is called the regularization matrix operator, and *λ* (positive scalar) is called the regularization coefficient or regularization parameters [102–110, 189–195].

Thus, the Gauss Newton method based minimization technique of EIT image reconstruction algorithm gives the conductivity update vector [Δ*σ*] as

(19) $$\begin{array}{c} \Delta \sigma =\frac{s_{r}^{\prime }}{s_{r}^{\prime \prime }}=\frac{{\left(F^{\prime }\right)}^{T}\left(V_{m}-F\right)-\lambda {\left(R\right)}^{T}\left(R\sigma \right)}{{\left(F^{\prime }\right)}^{T}\left(F^{\prime }\right)-{\left(F^{\prime \prime }\right)}^{T}\left(V_{m}-F\right)+\lambda R^{T}R}. \end{array}$$

Now, neglecting the higher order matrix [*f*′′]^*T*^ in (19), called the “Hessian matrix,” and replacing the matrix [*F*′] by the sensitivity matrix of the system [*J*], called the “Jacobian matrix” of the system, the conductivity update vector [Δ*σ*] reduces to

(20) $$\begin{array}{c} \Delta \sigma =\frac{J^{T}\left(V_{m}-f\right)-\lambda I\sigma }{J^{T}J+\lambda I}, \end{array}$$

where the matrix [*I*] represents the identity matrix which is equal to [*R* ^*T*^][*R*].

Therefore, the GN-EIRA of the EIT provides the general solution of the conductivity distribution of the DUT at the *k*th iteration of the algorithm as

(21) $$\begin{array}{c} \left[\sigma_{k+1}\right]=\left[\sigma_{k}\right]+{\left[{\left[J^{T}J+\lambda I\right]}^{-1}\left[J^{T}\left[V_{m}-f\right]-\lambda I\sigma \right]\right]}_{k}. \end{array}$$

### 7.13. Subject Under Test (SUT) in EIT

The subject to be imaged or subject under test (SUT) in EIT may be a human subject (healthy human volunteer or a patient) or laboratory animal or a tissue mimicking model object called “practical phantom” [117, 129, 131, 186, 188, 197–202]. The EIT instrument, called electrical impedance tomograph, is applied to the SUT and the EIT imaging is conducted for the domain of interest or domain under test (DUT) at a particular plane within the SUT in 2D or for a number of parallel 2D EIT domains called image slices in 3D EIT.

#### 7.13.1. Practical Phantoms

EIT phantoms [117, 129, 131, 186, 188, 197–202] which are the mimic models of the human body tissue or human body parts are essentially required to assess the performance of electrical impedance tomography (EIT) systems for their validation, calibration, and comparison purposes. EIT phantoms are classified mainly into two types, namely, network phantoms [201, 203–209] and real object phantoms [115, 186, 187, 198–200, 202, 210–215]. Real object phantoms are developed with a phantoms tank filled with two or more objects (biological or nonbiological) with different conductivities surrounded by an array of surface electrodes [97] housed on the inner wall of the phantom tank. Researchers have reported several types of real object phantoms for studying their EIT systems such as saline-insulator phantoms [115, 186, 187, 210–215], saline-agar phantom [216–218], saline-vegetable phantom [198, 212, 215, 219, 220], and passive or active element phantoms [200, 221]. All the phantoms have their own advantages and disadvantages. Network phantoms are developed with circuit elements housed on a electronic element base or printed circuit board (PCB). Network phantoms do not require any surface electrodes; only electronic connections are sufficient to interface the phantom with the EIT instrumentation. Though the network phantoms with electrical components are sometime advantageous for their long life, rigidity, stability, and ease of control, yet these phantoms fail to provide a mimic model the real biological tissue or the real body parts.

#### 7.13.2. Disadvantages of Saline Phantoms

Saline phantoms with solid inorganic (plastic, wood, or metal rod) or organic (vegetables or other biological tissues) materials are very popular in EIT as they are low cost and easy to develop and can be developed with rigid or flexible tanks of any shape and size. However, they cannot be assumed as a perfect mimic of the body parts as the background medium is a pure saline solution (purely resistive), whereas none of the human body parts are purely resistive in nature. Also, it is quite difficult to reconstruct the actual resistivity of the insulator inhomogeneity in a saline background because of their large resistivity difference. As the frequency versus impedance response of the saline solution is constant, the saline phantom fails to present a mimic model of body tissue as the response of the real tissue varies with frequency according to their physiological and physiochemical compositions contributing to a complex bioelectrical impedance. Moreover, the evaporation of the saline solution makes them unstable over the time which makes the assessment erroneous in real case. Hence the saline phantoms and the phantoms with circuit elements sometimes fail to calibrate the multifrequency EIT systems and the medical EIT systems. It is found that the practical biological phantoms consisting two different materials with low resistivity difference, such as the real tissue phantoms [188, 197, 200], are more suitable for multifrequency impedance imaging studies.

### 7.14. Scope and Challenges in EIT

Though the EIT technology has been studied in different fields of science and technology, yet EIT has shown its several advantages in medical imaging, and hence a number of research groups are working on it for medical and clinical imaging purposes. Although, due to poor signal to noise ratio (SNR) [222–225], of the boundary potential data and poor spatial resolution [226–228] the EIT technology has a lot of scope to work on it and a number of technical challenges to be solved. Therefore, though a medical EIT system has a number of advantages over the available regular medical imaging modalities, and the technical challenges and research scope of EIT technology have ever attracted the researcher to work for improving EIT reconstruction. A lot of research opportunities are still there in absolute impedance imaging, high speed reconstruction, improved 3D reconstruction, and spatial resolution of EIT.

## 8. Present Status and Future Directions of Impedance Methods

Multifrequency bioelectrical impedance analysis (BIA) [229, 230] is found as one of the core interest among the researchers in the field of bioelectrical impedance. A part of the researchers are studying on the instrumentation who are developing more sophisticated instrumentation. The future studies may be conducted on the wireless and Bluetooth based instrumentation for BIA techniques. Among the multifrequency impedance analyzing techniques EIS is the most popular and strong method. EIS methods are now being studied with a pulsed signal based EIS instrumentation [231–233] for tissue characterizations as well as single cell analysis [231–233]. The wireless and Bluetooth based instrumentation for EIS techniques can also be implemented in future studies. Presently the IPG is being studied for finger plethysmography [234, 235] by J. G. Webster group in University of Wisconsin, Madison, USA. J. G. Webster group is also working on the automated IPG instrumentation and the modern software based IPG systems [236–238]. In future, the wireless and Bluetooth based instrumentation for IPG techniques can also be implemented. ICG has several advantages in cardiac parameter assessment over the other conventional invasive methods. In recent years the ICG instruments are available from few industry-institute research collaborations. ICG can be studied as multifrequency bioimpedance methods for transthoracic parameter assessment for better cardiac health monitoring. ICG can also be studied as an ambulatory monitoring or long-term monitoring modality in intensive care unit (ICU). The electrical behavior of biological tissue is very complex, and hence, though the BIA, EIS, IPG, ICG are found with some successful applications, yet the EIT has not yet been considered as the regular medical imaging modality. Nonlinearity, ill-posedness, modelling error, measurement error, and other challenges are still required to be overcome. But, due to some unique advantages, if these challenges are overcome by future research on EIT, it may be also applied more effectively and efficiently compared to the other imaging modalities which are now being commonly used in some particular medical applications like brain imaging [239–241], breast imaging [242–249], abdominal imaging [250–252], whole body imaging [253–256], and so forth. Therefore, the EIT technology has a lot of potentials for low cost fast tomographic imaging, though the problems of low spatial resolution and poor signal to noise ration of the system should be solved in future research. Absolute conductivity imaging, better electrode performance, accurate system modelling, and high speed 3D EIT are also to be explored more to improve the EIT technology.

## 9. Conclusions

Bioelectrical impedance methods used for noninvasive health monitoring are reviewed in detail in this paper. The paper reviewed the technical aspects of some major bioimpedance methods such as bioelectrical impedance analysis (BIA), electrical impedance spectroscopy (EIS), electrical impedance plethysmography (IPG), impedance cardiography (ICG), and electrical impedance tomography (EIT). Detailed discussions on the theoretical aspects, working principles, applications, advantages, limitations and present research scenario, future trends, and challenges of these bioimpedance methods have been presented in detail in this paper. It is found all the methods are comparatively low cost, fast, portable, and simple. All the methods have a number of advantages in their application for the noninvasive investigations compared to the other methods, and hence they have been potentially applied for noninvasive diagnosis of tissue health. BIA, EIS, IPG, and ICG have been successfully studied by a number of researchers and scientists over past few decades for assessing a number of tissue parameters and the tissue compositions for tissue health diagnosis. BIA, EIS, IPG, ICG, and EIT have been used for biological tissue characterization by utilizing the electrical impedance information. BIA, EIS, IPG, and ICG study the tissue properties in terms of the lump impedance parameters at a particular frequency or at different discrete frequencies obtained from the boundary voltage current measurement. On the other hand the EIT provides a spatial distribution of the impedance profile of a 2D or 3D domain under test using a set of boundary voltage-current data. In this regard, EIT provides more information about the tissue physiology and pathology, and hence it has more potential in several applications. The paper presents a clear and detailed technical overview of BIA, EIS, IPG, ICG, and EIT methods and their applications for noninvasive tissue characterization and tissue health diagnosis which will help the readers to get the clear technical perspectives of these impedance analyzing techniques.

## Figures and Tables

**Table 1:** Body-composing constituents and their explanation and function [43].

Body-composing constituents	Explanation and function
Intracellular water (ICW)	Body water which exists inside of cell membrane

Extracellular water (ECW)	Body water that exists outside of cell membrane (blood, interstitial fluid, etc.)

Body water	The sum of intracellular and extracellular water

Protein	Main element which composes soft lean mass together with water

Soft lean mass (SLM)	Skeletal muscle and smooth muscle Maintaining body function

Minerals	Composing bones and electrolytes

Lean body mass (LBM)	Composed with soft lean mass and minerals The amount of body weight minus body fat mass

Body fat	The amount of body weight minus lean body mass

Weight	The sum of lean body mass and body fat mass. Standard weight (kg): adult male height (m) × height (m) × 22 female height (m) × height (m) × 22
